# The (extended) achondroplasia foramen magnum score has good observer reliability

**DOI:** 10.1007/s00247-022-05348-0

**Published:** 2022-04-09

**Authors:** Nathan Jenko, Daniel J. A. Connolly, Ashok Raghavan, James A. Fernandes, Shungu Ushewokunze, Heather E. Elphick, Paul Arundel, Utku Alhun, Amaka C. Offiah

**Affiliations:** 1 Department of Radiology, Sheffield Teaching Hospitals NHS Foundation Trust, Glossop Road, Sheffield, S10 2JF UK; 2 Sheffield Children’s Hospital NHS Foundation Trust, Sheffield, UK; 3 Department of Oncology & Metabolism, University of Sheffield, Sheffield, UK

**Keywords:** Achondroplasia, Achondroplasia foramen magnum score, Apnea, Children, Foramen magnum, Magnetic resonance imaging

## Abstract

**Background:**

Achondroplasia is the most common skeletal dysplasia. A significant complication is foramen magnum stenosis. When severe, compression of the spinal cord may result in sleep apnea, sudden respiratory arrest and death. To avoid complications, surgical decompression of the craniocervical junction is offered in at-risk cases. However, practice varies among centres. To standardize magnetic resonance (MR) reporting, the achondroplasia foramen magnum score was recently developed. The reliability of the score has not been assessed.

**Objective:**

To assess the interobserver reliability of the achondroplasia foramen magnum score.

**Materials and methods:**

Base of skull imaging of children with achondroplasia under the care of Sheffield Children’s Hospital was retrospectively and independently reviewed by four observers using the achondroplasia foramen magnum score. Two-way random-effects intraclass coefficient (ICC) was used to assess inter- and intra-observer reliability.

**Results:**

Forty-nine eligible cases and five controls were included. Of these, 10 were scored normal, 17 had a median score of 1 (mild narrowing), 11 had a median score of 2 (effacement of cerebral spinal fluid), 10 had a score of 3 (compression of cord) and 6 had a median score of 4 (cord myelopathic change). Interobserver ICC was 0.72 (95% confidence interval = 0.62–0.81). Intra-observer ICC ranged from 0.60 to 0.86. Reasons for reader disagreement included flow void artefact, subtle T2 cord signal and myelopathic T2 cord change disproportionate to canal narrowing.

**Conclusion:**

The achondroplasia foramen magnum score has good interobserver reliability. Imaging features leading to interobserver disagreement have been identified. Further research is required to prospectively validate the score against clinical outcomes.

## Introduction

Achondroplasia is the most common skeletal dysplasia with a prevalence of approximately 1 in 20,000 births. The condition is caused by mutations in the *FGFR3* gene and is inherited as an autosomal dominant mutation. Features of achondroplasia include disproportionate short stature with limb shortening, distinctive craniofacial features, kyphoscoliosis, accentuated lumbar lordosis and notably craniocervical junction stenosis [1].

Infants with achondroplasia have increased rates of both central and obstructive apnea [2]. Cord compression can result in sudden central apnea, potentially leading to cardiopulmonary arrest, hypoxic brain injury and, in certain cases, death. This hypothesis is supported by postmortem studies, where death has been attributed to severe craniocervical junction or upper cervical stenosis [3, 4].

In cases where craniocervical junction stenosis is identified, neurosurgical decompression of the craniocervical junction is thought to be effective in preventing apnea. Possible surgical complications include cerebrospinal fluid leak, craniocervical instability and postoperative infection. Stenosis of the craniocervical junction may recur, necessitating further surgery [5–8]. It is therefore vital to identify children who will benefit from neurosurgical intervention, while avoiding unnecessary intervention.

To date, insufficient data is available to produce evidence-based guidelines on selection for and optimal timing of craniocervical junction decompression surgery. The American Academy of Pediatrics advises that all children with achondroplasia be carefully assessed clinically, with sleep studies (polysomnography) and neuroimaging, which may include computed tomography (CT) or magnetic resonance imaging (MRI) [9]. Further research attempting to ascertain scientific consensus using the Delphi consensus process was published in 2015. Of note, there was consensus disagreement with the statement, “All children should undergo screening MR or screening CT” [10]. Oftentimes, MRI must be performed under sedation or anaesthesia, the risk of which must be balanced against the benefits.

Research on how to best utilize MRI to identify children who would benefit from neurosurgical intervention is ongoing. Practice varies among centres, with published intervention rates ranging from 4.5% [7] to 42.2% [11, 12].

The achondroplasia foramen magnum score was developed by Cheung et al. [13] to standardize the MRI assessment of the foramen magnum. The strengths of the score lie in the fact that it relies on standard T2 sagittal images and that it assesses the underlying neuroaxis at the craniocervical junction without relying on measurements. The score employs a 5-point (0 to 4) scale; 0 represents a normal foramen magnum, 1 represents a narrowed craniocervical junction with maintained cerebrospinal signal around the cord, 2 represents effacement of the cerebrospinal fluid signal at the craniocervical junction, 3 represents indentation of the cord at the craniocervical junction and 4 represents compression with myelopathic increased T2 cord signal. Authors of the score hope that a standardized scale will enable the creation of evidence-based guidelines, standardize practice and reduce morbidity [13].

Cheung et al. [13] developed the achondroplasia foramen magnum score on a data set of 36 patients; imaging was assessed by one of the authors (a paediatric radiologist); inter-observer variability was not evaluated. In this work, we assess the interobserver variability of the score on a different and larger patient cohort. The secondary aims of the study were to assess the correlation between the achondroplasia foramen magnum score, MR cerebrospinal fluid flow studies and findings on polysomnography.

## Materials and methods

Children with achondroplasia, who are or had been under the care of Sheffield Children’s Hospital and had base of skull MR scans, were identified by searching the Picture Archiving and Communications system (PACS). The PACS data was correlated with clinic lists and sleep study results. Where multiple studies were available for assessment, the first study was selected.

At our institution, MR imaging is initially attempted using a “feed-and-wrap” method. If the obtained imaging is judged to be too degraded (e.g., by movement), a repeat study under general anaesthesia is performed. To enable comprehensive assessment of the craniocervical junction, the protocol includes sagittal T2 imaging, high-resolution axial imaging of the craniocervical junction and cerebrospinal fluid flow cine studies. Additionally, standard T2 and proton density sequences of the brain are obtained. We also routinely obtain T1 and T2 imaging of the whole spine.

Studies from referring district general hospitals included sagittal and axial T2 imaging with additional high-resolution sequences often being performed. Digitized patient records, sleep study results and clinic letters were used to record other relevant findings.

MR imaging was independently assessed by four observers. These were two specialist paediatric neuroradiologists (A.R., D.J.A.C.), who regularly report these studies; a specialist paediatric musculoskeletal radiologist with extensive knowledge of skeletal dysplasias (A.C.O.) and a 3^rd^ year (United Kingdom [UK] specialty training, third year [ST3], post-graduate year four equivalent) radiology registrar undergoing a paediatric neuroradiology specialist placement (N.J.). The consultants each have more than 15 years of experience in post.

Images of five (9.3%) additional patients without achondroplasia were included as controls.

**Fig. 1:**
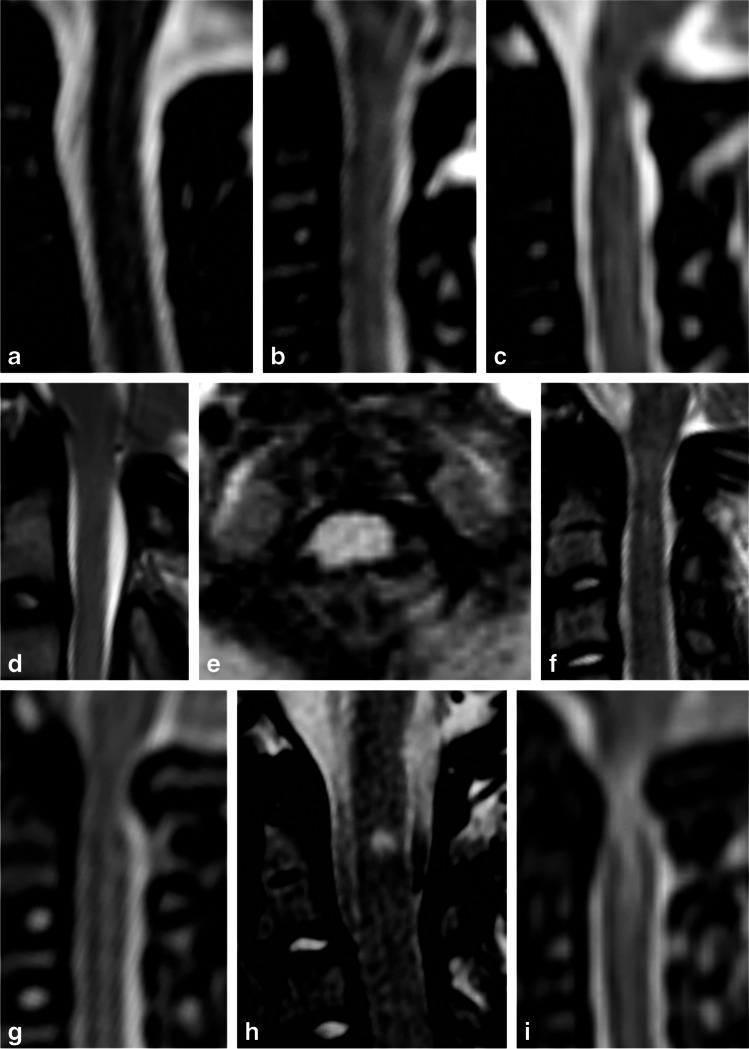
Representative T2 sagittal imaging showing eAFMS; see Table 1 for a description of the findings in each grade. Sagittal (**a-d, f-i**) and axial (**e**) T2 magnetic resonance images: **a** A 23-month-old girl with a normal CCJ. **b** A 30-month-old girl with AFMS 1 stenosis. **c** An 8-month-old girl with AFMS 2a stenosis. **d** A 4-year-old boy with AFMS 2b stenosis. **e** An 8-month-old girl with AFMS 2c stenosis. **f** A 7-year-old girl with AFMS 3a stenosis. **g** A 12-month-old boy with AFMS 3b stenosis. **h** A 7-year-old girl with AFMS 4a stenosis. **i** A 9-month-old boy with AFMS 4b stenosis. *AFMS* achondroplasia foramen magnum score, *CCJ* craniocervical junction*, eAFMS* extended achondroplasia foramen magnum score

Imaging was assessed using the published achondroplasia foramen magnum score [13]. The observers also scored the images using an extended score, reflecting the level of detail local reports would normally provide (Table 1 and Fig. 1). Observers assessed all cases using the 5-point achondroplasia foramen magnum score both before and after the development of the extended score. To ensure intra-observer independence of both observations, a period of 8 months elapsed between them. The intra-observer correlation coefficient was calculated by mapping the extended score from the second reads onto the original achondroplasia foramen magnum score; the intraclass coefficient (ICC) was then calculated between the first and second scores for each observer.

**Table 1:** The achondroplasia foramen magnum score (AFMS) and extended AFMS (eAFMS) scores

**Score**	**AFMS**		**eAFMS**
0	Normal appearances of the craniocervical junction	0	Normal appearances of the craniocervical junction
1	Mild narrowing of the craniocervical junction	1	Mild narrowing of the craniocervical junction
2	Effacement of CSF signal at the craniocervical junction	2a	Narrowing of the craniocervical junction, which effaces CSF signal posterior to the cord
		2b	Narrowing of the craniocervical junction, which effaces CSF posteriorly and anteriorly
		2c	Narrowing of the craniocervical junction, which effaces CSF circumferentially
3	Indentation of the cervical spinal cord	3a	Remodelling (visible indentation) of the cervical spinal cord, CSF signal remains present
		3b	Remodelling (visible indentation) of the cervical spinal cord, CSF signal is effaced
4	Myelopathic T2 signal change in the cervical spinal cord	4a	Myelopathic T2 signal change, CSF signal remains present
		4b	Myelopathic T2 signal change, CSF signal is effaced

*CSF* cerebrospinal fluid

Polysomnography results were analysed using criteria from American Academy of Sleep Medicine [14]; the Apnea and Hypopnea Index (AHI) was calculated, and events were further classified into central and obstructive events.

Data was analysed using Stata Statistical Software (Release 13, StataCorp LP, College Station, Texas). Due to an even number of observers, some median scores (17 patients, 31.5%) were halfway between grades. In those cases, the scores from a fifth observer (a further UK ST3 registrar, U.A.) were included to obtain whole scores. Observer reliability data (achondroplasia foramen magnum score) was analysed using two-way random-effects ICC (2,1) [15].

Patient/parental consent and formal research ethics approval were not required for this retrospective review of images. The study was approved and registered with the local Service Evaluation Department prior to commencement.

## Results

In total, 85 patients were identified, 49 of whom had an appropriate MRI available. All postsurgical imaging was excluded. With the addition of 5 controls, a total of 54 studies were included. Where there were multiple studies available for a patient, the first study of diagnostic quality was scored. The median age of included children was 21 months (interquartile range [IQR] = 6–85 months).

Median achondroplasia foramen magnum scores are the following: In 10 (18.5%) patients (median age: 18.5 months, IQR = 2–42), the craniocervical junction was scored as normal; in 17 (31.5%) patients (median age: 64 months, IQR = 21–125), there was mild narrowing of the craniocervical junction (Grade 1); in 11 (20.4%) patients (median age: 32 months, IQR = 8–70), there was effacement of the cerebrospinal fluid signal (Grade 2); in 10 (18.5%) patients (median age: 13 months, IQR = 5–40), there was indentation of the cervical cord (Grade 3); and, in 6 (11.1%) patients (median age: 9.5 months, IQR = 8–11), myelopathic T2 signal change was identified in the spinal cord (Grade 4). Of the normal controls, four (80%) were scored as normal and one (20%) was scored as mildly narrowed (Grade 1).

The ICCs for the achondroplasia foramen magnum stenosis and extended foramen magnum scores were 0.72 (95% confidence interval [CI] = 0.62–0.81) and 0.72 (95% CI = 0.62–0.82), respectively, which is normally interpreted as good agreement [16]. The scores provided by the registrar did not significantly differ from the consultant scores; when the registrar’s scores are excluded, the ICC is 0.68 (95% CI = 0.55–0.79).

**Fig. 2:**
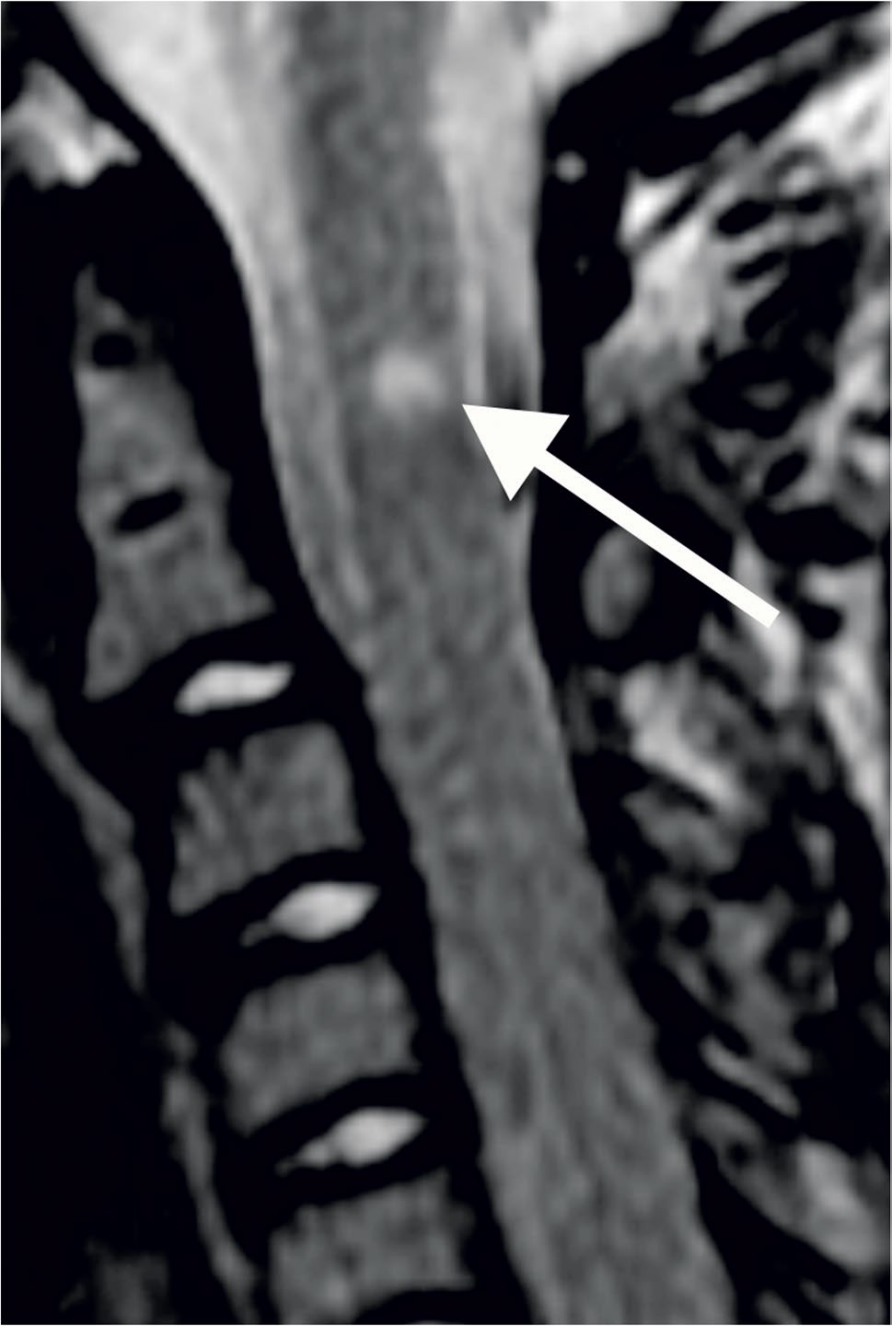
A sagittal T2 magnetic resonance image of the craniocervical junction in a 7-year-old girl with achondroplasia. T2 signal change is present in the cervical cord (*arrow*) without evidence of foramen magnum stenosis

**Fig. 3:**
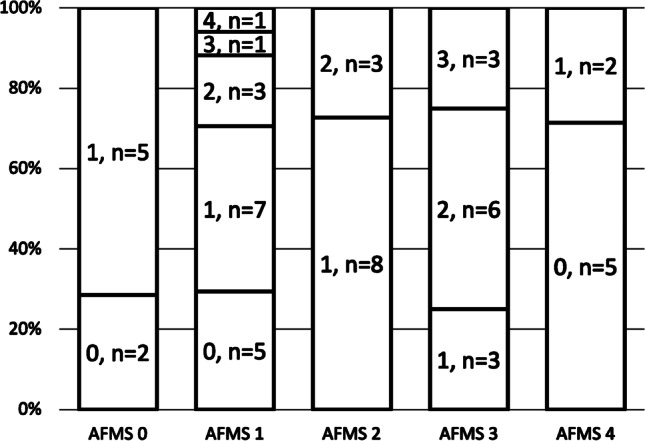
Difference in observer scores for each achondroplasia foramen magnum score grade. 0 = full agreement; 1 = 1-point disagreement; 2 = 2-point disagreement; 3 = 3-point disagreement between observers

For 12 (22.2%) studies, there was complete agreement between the observers; in 25 (46.3%) studies, there was 1 point of maximum disagreement between the observers; in 12 (22.2%) studies, there was 2 points of maximum disagreement and in 4 (7.4%) studies, there was a maximum disagreement of 3 points. There was one study rated as showing severe stenosis (Grade 4) by one observer but as not significantly narrowed (Grade 0–1) by the other observers; in this case, there was 4 points of maximum disagreement (Fig. 2). A graphical summary of the findings in this paragraph is presented in Fig. 3.

The intra-observer ICCs (95% CI) for the 4 observers were: 0.86 (0.76–0.92), 0.80 (0.65–0.88), 0.63 (0.33–0.80) and 0.60 (0.38–0.77). This is interpreted as good to excellent agreement [17].

**Fig. 4:**
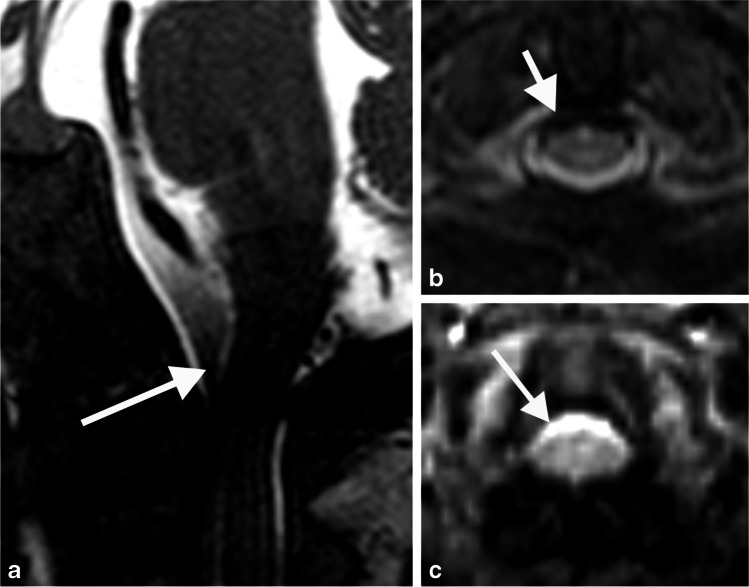
The effect of cerebrospinal fluid (CSF) flow void on achondroplasia foramen magnum score (AFMS): Sagittal (**a**) and axial (**b, c**) magnetic resonance images of the craniocervical junction in a 4-month-old girl with achondroplasia. Normal high signal CSF is seen above and below the foramen magnum. CSF flow void (*arrows*) anterior to the spinal cord is indistinguishable from the cortex of adjacent bone on T2 sequences (**a, b**). In contrast, CSF signal (*arrow*) is clearly present on the gradient echo sequence (**c**). CSF flow void (**a, b**) may lead to the assignment of a higher AFMS score than is warranted

**Fig. 5:**
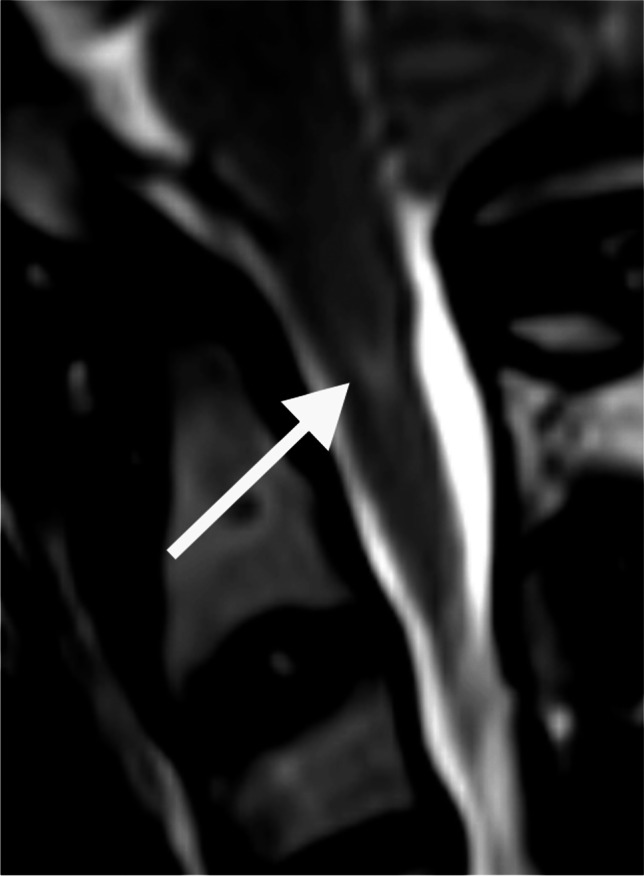
Sagittal T2 magnetic resonance images of the craniocervical junction in a 12-year-old girl with achondroplasia. There is subtle increased T2 signal (*arrow*) without evidence of foramen magnum stenosis

After review of the MRI studies for which there were at least three points of disagreement between observers, three causes were identified: large cerebrospinal fluid flow voids obscuring bone detail (Fig. 4), subtle increased T2 signal in the spinal cord (Fig. 5) and myelopathic T2 signal change disproportionate to canal narrowing (Fig. 2).

**Fig. 6:**
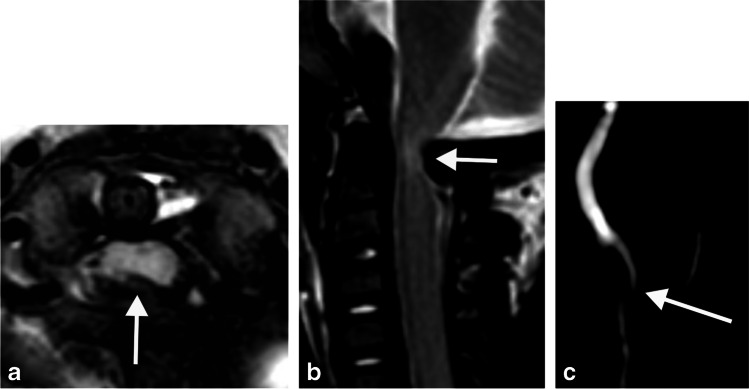
Cerebrospinal fluid flow despite central spinal cord compression in an 11-month-old girl. Axial T2 (**a**), sagittal T2 (**b**) and phase contrast Cerebrospinal fluid (CSF) flow sequence (**c**) shows central hypertrophy of the foramen magnum (*arrows*) compressing the spinal cord (**a, b**) and CSF flow anteriorly and laterally to the cord on phase contrast imaging (**c**)

Cerebrospinal fluid flow using a phase contrast technique [17] was incorporated in 11 MRI studies. These included four with an achondroplasia foramen magnum score of 3; in three of these four cases, cerebrospinal flow was detected at the craniocervical junction. There were three cases with an achondroplasia foramen magnum score of 4; in one of these three cases, cerebrospinal fluid flow remained (Fig. 6).

Only two infants underwent preoperative polysomnography; polysomnography was insufficient to exclude significant cord compression. Eight children underwent surgical decompression; the achondroplasia foramen magnum score results for these cases are provided in Table 2.

**Table 2:** Findings in patients who underwent craniocervical junction decompression

Patient	Age at imaging (months)	Age at surgery (months)	Median AFMS score	MR cine CSF flow
1	3	3	4	Not performed
2	6	6	3	Not performed
3	7	7	4	CSF flow absent
4	1	10	3	Not performed
	9		4	CSF flow present
5	9	10	4	CSF flow absent
6	10	10	4	Not performed
7	11	11	4	CSF flow present
8	31	34	4	Not performed

*AFMS* achondroplasia foramen magnum score, *CSF* cerebrospinal fluid, *MR* magnetic resonance

## Discussion

Sheffield Children’s Hospital accepts referrals from the South Yorkshire region in the United Kingdom and provides specialist paediatric care to a population of approximately 2 million. MRI is often performed at the patient’s local hospital and the images in this study therefore represent a pragmatic “real-life” data set, sourced from different regional hospitals using different equipment and protocols. For clinical purposes, we routinely provide a descriptive report for MRI studies of the craniocervical junction and noted with interest the introduction of an achondroplasia foramen magnum score [13]. However, the publication did not indicate interobserver reliability of the score and neither (as far as we are aware) has any other group assessed this important parameter. Based on one of the largest data sets published in relation to foramen magnum stenosis in achondroplasia, we found that among four radiologists, the interobserver correlation coefficient of the 4-point score was 0.72, signifying good agreement [16].

The ICC of the achondroplasia foramen magnum score in this current study (0.72) is comparable to that of the cervical stenosis score developed for use in adults (ICC = 0.77) [18] and to scoring systems in widespread use, such as the Liver Reporting & Data System (LI-RADS) MRI score for the assessment of hepatocellular carcinoma (ICC = 0.73) [19] and Prostate Imaging Reporting & Data System (PI-RADS) for the assessment of prostate cancer (ICC = 0.71) [20]. It is hoped that standardizing the imaging protocol throughout the referral network, increased experience with the score, and widespread use of 3-T scanners will improve the ICC of the achondroplasia foramen magnum score.

One of the causes of interobserver disagreement was large cerebrospinal fluid flow voids obscuring bone detail. Cerebrospinal fluid flow voids occur due to protons moving out of plane between spatially selective radiofrequency pulses and appear as a lack of signal on the MRI. Cerebrospinal fluid flow voids occur normally at the craniocervical junction but can appear particularly prominent if the craniocervical junction is narrowed, causing an increase in the velocity of the cerebrospinal fluid. The flow void may become inseparable from the adjacent bony contour of the foramen magnum (Fig. 4). Gradient-echo based sequences use a shorter delay between the excitation and the refocusing pulse and are hence less susceptible to flow voids.

The achondroplasia foramen magnum score presumes a logical sequential progression of features consistent with increasing spinal cord compression. Effacement of cerebrospinal fluid signal is followed by cord indentation, which then results in myelopathic cord signal change. However, our data set does not fully support this logical sequence, containing three studies where cerebrospinal fluid signal is maintained despite indentation of the cord (achondroplasia foramen magnum score 3a example image, Fig. 1). Similarly, there were two studies showing presumed myelopathic T2 change despite maintained cerebrospinal fluid signal (Fig. 2). The use of the extended achondroplasia foramen magnum score would accurately capture these cases. Of note, extending the score, while increasing the descriptive assessment, did not introduce any additional interobserver variance, with the ICC remaining at 0.72.

A previous study [21] in an older achondroplasia population (40.7 ± 15.3 years) found that 7 of 18 (39%) patients had areas of high T2 signal in their spinal cervical cord (“cervical high-intensity intramedullary lesions”) and that in 6 of the 18 cases (33%) indentation of the cervical cord without external compression was present. These findings were not associated with clinical symptoms. The authors were unable to elicit positional (flexion/extension) compression in adulthood but suggest the findings may be due to earlier, now resolved, positional compression. We did not identify any cases of infants with increased T2 signal in the cord and maintained surrounding cerebrospinal fluid space but did observe this pattern in two children ages 7 years and 12 years (Figs. 2 and 5). However, cord indentation with maintained cerebrospinal fluid signal was visible earlier in patients ages 5 months, 8 months and 3 years. A previous case series [8] has shown that positional compression is common with 38% of studied infants exhibiting stenosis evident on dynamic flexion/extension imaging. It is possible that indentation followed by high T2 signal with maintained cerebrospinal fluid signal is the natural progression of the imaging appearances of positional compression. There is no consensus on the management of positional cord compression [10] and the risk of adverse respiratory events has not been established. Distinguishing patients with high cord signal (Grade 4) or indentation (Grade 3) who do have cerebrospinal fluid flow around the cord from those who do not is one of the benefits of the extended achondroplasia foramen magnum score.

Phase-contrast cerebrospinal fluid flow studies were only available for seven patients. Of note is the fact that even with imaging showing indentation of the cord (in four of these seven studies), flow remained present at the craniocervical junction. In 1987, work on CT imaging of the foramen magnum identified hypertrophy of the posterior occiput as a key contributor to cervical spinal cord compression [22]. The imaging findings in our data set confirms that the spinal cord can be compressed centrally by occipital hypertrophy, while cerebrospinal fluid flow remains present laterally in the foramen magnum (Fig. 6).

**Fig. 7:**
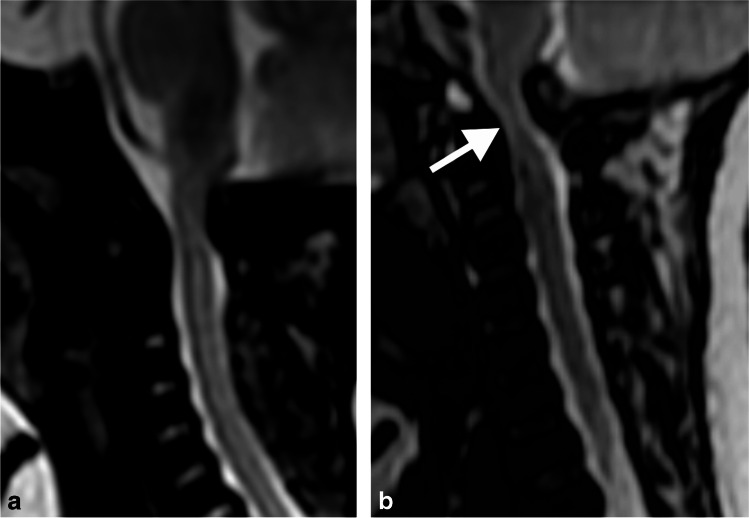
Sagittal T2 magnetic resonance images of the craniocervical junction in a girl with achondroplasia at 1 month (**a**) and 9 months (**b**) of age. The images show interval progression of stenosis from Grade 3 to Grade 4 due to occipital hypertrophy, which indents the spinal cord (*arrow*)

Ideally, MRI should be obtained when the child is 6–12 months old, when the size of the foramen magnum has been shown to be at its narrowest, due to a combination of premature posterior synchondrosis fusion and impaired growth [22]. In one patient in our cohort, imaging was initially performed at 1 month, then repeated at 9 months of age. The latter demonstrated interval progression of cord compression in line with the achondroplasia foramen magnum score progressing from 3 to 4 (Fig. 7). At our institution, we do not routinely arrange for follow-up MRI after 12 months of age, unless there is a clinical indication.

The consensus guidelines state that cord change (Grade 4) is an indication for surgical decompression of the craniocervical junction [10]. This is consistent with the practice at our centre, where all patients with MR-demonstrated compression and myelopathic cord signal change were decompressed – five of the patients had presented with episodes of desaturation, one patient developed new clonus and one patient underwent surgery electively based on MR findings. Cases with indentation of the spinal cord (Grade 3) were assessed clinically, with one of the four patients proceeding to decompression; clinically, the patient was found to have decreased tone, increased reflexes and reported episodes of desaturation. Overall, the intervention rate in our centre was 14.8%, which is near the median of published figures ranging from 4.5% to 42.6% [7, 11, 12].

Multiple case series [11, 13, 23] have previously argued for universal assessment of the craniocervical junction in infants with achondroplasia using MR noting that polysomnography is not sufficiently sensitive. In our cohort, two infants underwent preoperative polysomnography. One infant (Patient 3, Table 2) underwent polysomnography preoperatively after inadequate imaging was obtained using the feed-and-wrap method. The initial sleep study was mildly abnormal (mixed obstructive and central events, AHI = 10.4); a repeat study was performed, which was normal (AHI = 0.6). The patient presented 9 days later, at the age of 7 months, with respiratory arrest; severe stenosis in keeping with Grade 4 (Fig. 7) was identified on MRI. The patient then underwent surgical decompression of the craniocervical junction. Our limited evidence agrees with previous studies that polysomnography cannot be relied upon to detect cord compression in infants with achondroplasia.

The limitations of this study are its retrospective nature, limited patient numbers and sourcing of data from a single referral network. Future work is required to correlate imaging findings to clinical outcomes, perhaps using a multicenter case–control study design, stratified by center, as a randomised controlled trial would not be ethical.

## Conclusion

The achondroplasia foramen magnum score has good intra- and interobserver agreement (ICC = 0.72), comparable to the ICC of other scoring systems in clinical use. The score should be prospectively validated against clinical outcomes.
